# The Pacific Island Health Care Project

**DOI:** 10.3389/fpubh.2014.00175

**Published:** 2014-10-13

**Authors:** Donald Ames Person

**Affiliations:** ^1^Tripler Army Medical Center, Honolulu, HI, USA

**Keywords:** telemedicine, teleconsultation, tele-referral, Pacific Island, Pacific Islands Healthcare Project

## Abstract

**Introduction/Background:** US Associated/Affiliated Pacific Islands (USAPI) include three freely associated states: Marshall Islands, Federated States of Micronesia, Palau, and three Territories: American Samoa, Guam, and Commonwealth of the Northern Mariana Islands.

**Objective:** The Pacific Island Health Care Project (PIHCP) provides humanitarian medical referral/consultation/care to >500,000 indigenous people of these remote islands.

**Methods:** In the mid-1990s, we developed a simple store-and-forward program to link the USAPI with Tripler Army Medical Center. This application allowed image attachment to email consultations.

**Results:** More than 8000 Pacific Islanders have benefited from the program. Three thousand Pacific Islanders prior to telemedicine (1990–1997) and since store-and-forward telemedicine (1997-present), the PIHCP has helped an additional 5000. Records post dynamically and are stored in an archival database.

**Conclusion:** The PIHCP is the longest running telemedicine program in the world delivering humanitarian medical care. It has bridged the Developing World of the remote Pacific Islands with advanced medical and surgical care available at a major US military teaching hospital. (The opinions expressed here are those of the author and not that of the Army, Department of Defense, or the US Government.)

## Introduction/Background

The former US Pacific Trust Territories include Republic of the Marshall Islands, Federated States of Micronesia (FSM) (Chuuk, Kosrae, Pohnpei, and Yap States), and Republic of Palau. American Samoa in the South Pacific, Guam, and Commonwealth of the Northern Mariana Islands (CNMI) make up the remainder of the US Associated/Affiliated Pacific Islands (USAPI). American Samoa, CNMI, and Guam are US Territories. These remote nations, home to about 500,000 indigenous persons, include over 2200 islands and atolls scattered across more than 7 million square miles of Pacific Ocean (Figure S1 in Supplementary Material). These countries are part of the developing world of the Pacific, several lacking adequate infrastructure, transportation, communications, and medical support services. The people live in few of the islands and atolls of Micronesia in small family units. Many survive as subsistence fishers and farmers. Historically traditional healers, village elders, a few expatriate physicians, and nurses provided medical care to the people. An Institute of Medicine study detailed problems of medical care and referral from the US Pacific (1). The senior leadership (commander, department chairs, teaching chiefs, medical education directors) at Tripler Army Medical Center (TAMC) located in Honolulu, Hawaii had long recognized the medical need and unique teaching value of Pacific Islanders to our mission to provide for graduate medical education (GME). With training programs in surgery, medicine, pediatrics, psychiatry, otolaryngology (ENT), urology, orthopedics, and pathology it was incumbent on the leaders to find a way to organize, fund, and regulate the flow of patient cases from the Pacific. With the granting of full sovereignty to the jurisdictions in 1986, there was an influx of sick patients sent to TAMC from the islands. The Compact of Free Association provided for support of health and welfare in the jurisdictions and the provision of limited medical care at TAMC, as available, for selected Pacific Islanders on a reimbursable basis. The jurisdictions of the US Pacific are very poor and even partial reimbursement was unreasonable in the 1980s, much less now! In 1988, several of us began to discuss possible solutions to provide much-needed humanitarian care to Pacific Islanders at TAMC and at the same time enhance GME. Federal medical facilities neither make a profit nor provide charity. We presented our proposal to the late US Senator Daniel K. Inouye, an ardent supporter of Army medicine and a champion of the underserved peoples of the US Pacific, and he provided the means. Under his aegis, the US Congress appropriated funds for the travel of a limited number of Pacific Islanders (100 patients annually) to/from TAMC for medical care. TAMC bore all medical costs associated with the program. The Pacific Island Health Care Project (PIHCP) accepted its first patients in mid-1990. Almost since its inception, I have directed the program. Major referral sites included Majuro Hospital, state hospitals in Pohnpei, Chuuk, Kosrae, Yap, and Belau National Hospital, Palau. Physicians in the islands made referrals by mail, telephone, and facsimiles. Distance, an International Date Line, five time zones, unreliable communications, and language differences posed substantial problems. The tremendous cost of caring for hundreds of sick Pacific Islanders (300–450/year) threatened continuation of the program. In-patient costs of $50,000–$100,000/patient were commonplace. Difficulties with communication, misunderstanding of program limitations, etc., added to the confusion. Patients with disseminated malignancies, multiple congenital anomalies, heart failure, active tuberculosis, end-stage renal disease, stroke, etc., arrived at TAMC unannounced. Some patients died en route, others arrived *in extremis*. Despite these problems, nearly 3,000 Pacific Islanders were cared for under the PIHCP in the early years of the program and prior to telemedicine.

## Objectives

The PIHCP was developed to (1) provide humanitarian medical care to deserving, underserved, indigenous peoples of the USAPI at a reasonable cost, restore them to health, and return them to their island homes as productive members of society and (2) select patients to enhance GME for TAMC’s residents-in-training and staff (i.e., good teaching cases).

## Design/Methods

Real-time (synchronous) medical consultations made between Kwajalein Headquarters (US Army Missile Defense Command located in the Marshall Islands) and TAMC as early as 1992 using the AT&T Video teleconferencing system validated the ease of consultation (2). The utility of the Picasso telephone, demonstrated by me at the inaugural meeting of the Pacific Basin Medical Association (PBMA) in Pohnpei in 1995, confirmed that store-and-forward (asynchronous) consultation was just as easy to use (3) (Figure S2 in Supplementary Material). Excessive cost and needed technical expertise precluded the sustainability of either of those systems in the Pacific. Electronic mail with attached images provided a suitable medium for patient consultation and referral. With the technical assistance of Project Akamai, I was able to develop a simple store-and-forward method of telemedical consultation/referral. During the fourth Annual Meeting of the PBMA in Weno, Chuuk (1998), we demonstrated the utility of the Internet-based consultation/referral platform. Early results from four test sites confirmed the ease and utility of the web-based system (4, 5). TAMC’s judge advocate (senior attorney) and I discussed legal ramifications of PIHCP telemedicine program many times and decided that a consent statement/form be added to each patient’s consultation page. Indigenous island people do not speak English, so it was incumbent on the referring provider to assure patient understanding and consent.

## Results

We established 10 workstations in the USAPI between February 1998 and January 2001 (Table S1 in Supplementary Material). Fifty percent of referred cases (5422) came from Palau and the Marshalls (Table S2 in Supplementary Material). Males and females are equally represented, a quarter <18 years, a third >50 years, and the remainder, 18–50 years of age (Table S3 in Supplementary Material). Table S4 in Supplementary Material lists the departmental referrals. The majority of patients were referred to the Department of Surgery (general/oncologic, pediatric, vascular, plastics/reconstructive, neurosurgery, cardiothoracic, etc.). Subspecialty medicine referrals were next most frequent followed by “other” that includes ENT, ophthalmology, urology, radiology, dental, psychiatry, and pathology. Pediatric referrals were next. Gynecological malignancies made up the bulk of the cases referred to the Department of Obstetrics/Gynecology. Historically, 792 users had access to the website. Currently, there are 158 active users: American Samoa (5) Chuuk (5), CNMI (5), Ebeye (7), Guam (4), Kosrae (2), Majuro (14), Palau (17), Pohnpei (7), Yap (7), and 85 from the US. Except for a few nurses and administrators, all TAMC users are specialists or subspecialists. Island referring physicians are mostly generalists, some with additional certifications in surgery, medicine, pediatrics, or obstetrics/gynecology. A statistical module posts all data dynamically. All cases (patient web pages) with attached imagery (photographs, EKGs, x-rays, CT and MRI scans, laboratory results, histopathologic reports, echocardiograms, full motion video, etc.) are archived and retrievable (6, 7). Currently, about 20 cases are submitted monthly, during the early years, 30/month. The database is searchable using key words, terms, diagnoses, and phrases.

## Vignettes from the PIHCP

Diseases, once common in developed countries 50–100 years ago, but now mostly eradicated, are still prevalent in Oceania. Infectious diseases, such as tuberculosis, leprosy, rheumatic fever, leptospirosis, dengue, tropical pyomyositis, and necrotizing fasciitis, are common. Some unique conditions relate to culture and environment, such as billfish injuries, betel nut cancers, marine envenomations, motor boat injuries, burns, electrocution, are examples. Pacific Islanders also suffer from diseases associated with modern living; hypertension, diabetes, hyperlipidemia, gout, obesity, coronary artery disease, and cancer. Store-and-forward telemedicine is uniquely suited to consultation and referral from remote, resource poor areas of the developing world. In the early days of the program, few of the jurisdictions had more than antiquated x-ray machines. Film/developer was (and still is) often unavailable. There were no obstetrical ultra sound machines, much less CT scanners! Currently, three jurisdictions have CT scanners (Palau, Majuro, and American Samoa). Guam and Saipan (CNMI) have advanced and sophisticated imaging capabilities including CT and, MRI.

### Otolaryngology/head and neck surgery (ENT)

Common ENT referrals from the Pacific Islands include children with cleft lip/palate (Figure S3 in Supplementary Material). Oral cancers are especially prevalent in Palau, Yap, and Pohnpei, Islands where both men and women chew betel nut mixed with tobacco and slaked lime. Surgical management of these conditions provides ENT and maxillofacial residents with the skills necessary to treat injuries common in the war zone. A young boy from Kosrae fell on a coconut husker (spike/adze buried in the ground) and lacerated his trachea. Total body emphysema ensued and threatened his life. Our pediatric ENT surgeon performed a tracheostomy that allowed the air to dissipate. The child returned home a week later, cured (Figure S4 in Supplementary Material).

### Urology

Renal stones (nephrolithiasis), common in Pacific Islanders are easily treated with lithotripsy, unavailable in the islands. Large staghorn calculi, however, require surgical removal and provide excellent resident training. Rare male genital malignancies provide staff and residents with unique learning opportunities. TAMC’s urogynecologist collaborates with reconstructive urologists on complicated female cases. Hypospadias, epispadias, exstrophy of the bladder, cryptorchidism, and ambiguous genitalia challenge surgeons, as well as pediatric endocrinologists. We identified a Yapese family several years ago where three children with ambiguous genitalia had congenital adrenal hyperplasia. We reported a child from Chuuk with ambiguous genitalia that developed two malignancies (Li-Fraumeni syndrome) (8). A Chuukese boy fell and lacerated his perineal urethra. The photographic images attached to his case allowed our pediatric urologist to recommend temporizing measure (suprapubic cystostomy/wound care) prior to definitive repair (Figure S5 in Supplementary Material). Based on supporting images urology consultants are able to determine which cases to treat robotically.

### Orthopedics

Traumatic injuries, chronic fractures and dislocations, developmental anomalies, chronic osteomyelitis, musculoskeletal tumors, tropical pyomyositis, arthritis, spondylitis (including Potts disease), disk disease, and spinal stenosis are common in Pacific Islanders. Musculoskeletal cancers, uncommon in the US are frequent in Pacific Islanders. The treatment of patients with osteosarcoma, chondrosarcoma, Ewing sarcoma, rhabdomyosarcoma (Figure S6 in Supplementary Material), giant-cell tumors, aneurysmal bone cysts, and other rare musculoskeletal tumors enhance resident training. Photos, x-rays, ultra sound, and CT images enhance consultations. A chief orthopedics resident published our experience, highlighting the educational benefits to the orthopedic program of the PIHCP (9). These patients provide a wealth of experience for orthopedic residents and many of the skills learned are directly applicable to combat requirements.

### Cardiothoracic surgery

Acute rheumatic fever and rheumatic heart disease (RHD) occur frequently in children and young adults from the islands. Recurrent group A, β-hemolytic streptococcal infections lead to the development of valvular heart disease. Utilizing telemedicine, we can select those patients who would likely benefit from valve replacement surgery (compliance with penicillin prophylaxis). Most islanders have bioprosthetic valves placed. A pediatric resident and I recently reviewed and published our experience with RHD in Pacific Islanders (10). These consultations are enhanced with photographs (cyanosis, clubbing, anterior chest bulge) chest x-rays, EKGs, and ECHO video clips.

### Neurosurgery

Neurosurgical consultation is helpful and lifesaving in selected cases. Patients with myelomeningocele, hydrocephalus, paralysis, stroke, chronic and degenerative nervous system disease, etc., are not accepted. Long-term care and rehabilitation is not available. Our neurosurgeons have used endoscopic third ventriculostomy in selected cases and radiosurgery (gamma knife) in a few others. Treating patients with lesions amenable to neurosurgical intervention can be challenging but rewarding (11). Photographs, x-rays, CT scans, head ultrasound images, and full motion video clips attached to the consultation can be diagnostic.

### General, oncologic, pediatric, plastic, and vascular surgery

Our surgical training programs probably benefit the most from referrals from the USAPI. Several years ago, we consulted on a young Marshallese woman with recurrent hypoglycemia and a breast tumor. Her cystosarcoma phyllodes tumor secreted an insulin-like factor and mastectomy resulted in normalization of her blood sugar (12) (Figure S7 in Supplementary Material). In another remarkable case, surgical oncologists and orthopedic surgeons removed a massive osteosarcoma from the right shoulder a woman from Kapingamarangi (outer island in Pohnpei State) [Ref. (13), Figure S8 in Supplementary Material]. Pediatric surgeons treat children from the Pacific with diaphragmatic hernias, Hirschsprung’s disease, tumors [Ref. (14, 15), Figures S9 and S10 in Supplementary Material], congenital anomalies, and acquired conditions. Minimally invasive surgical techniques employed in recent cases allows for rapid recovery and short hospitalizations.

### Obstetrics and Gynecology

Telemedicine is especially useful in assessing patients with gynecological malignancies. Women with advanced, but treatable uterine and ovarian cancers provide a wealth of experience for our OB/Gyn training program (16). Women with gestational trophoblastic malignancies including choriocarcinoma, hydatidiform mole, and molar pregnancies have also benefited from the PIHCP. In some cases, consultant advice is all that is necessary. A pregnant Chuukese woman had a 0.22 caliber bullet lodged in her frontal lobe. Based on the teleconsultation, both neurosurgeon and perinatologist agreed that operative removal was contraindicated (Figure S11 in Supplementary Material). Sadly, shortly before delivery at home she suffered a grand mal seizure, delivered a stillborn, and died. Another pregnant woman with a massive umbilical hernia and peritoneal tuberculosis (tabes mesentarica) was consulted because of concerns for herniation and torsion of her gravid uterus. Photographs of her abdomen provided TAMC consultants (general surgeon and perinatologist) the information needed to assure the remote provider that it was safe to treat her tuberculosis and let her continue with her pregnancy. In another case, a 38-year-old Marshallese woman referred to TAMC had a 90-pound ovarian serous cystadenoma removed by our gynecological surgeons (Figure S12 in Supplementary Material).

## Discussion/Conclusion

Internal medicine and pediatric consultants are often the first to evaluate patients referred to the PIHCP. Many adult patients have significant comorbidities and successful surgical treatment depends on careful selection and pre-operative, perioperative control of factors such as hypertension, diabetes, renal insufficiency, hyperuricemia, hypoalbuminemia, hypokalemia, and other electrolyte disturbances, prior to and during surgery. Pre-operative dental extractions/restorations have contributed to the long-term success of our open-heart program. Infectious diseases, such as tuberculosis, leptospirosis, and leprosy are still prevalent in the Pacific Basin and prior to telemedicine; children with various forms of extra pulmonary tuberculosis (arthritis, osteomyelitis, spondylitis, mastoiditis, meningitis, etc.) came to TAMC for treatment. Such patients flew into Honolulu and presented in the TAMC emergency department causing disruption, delays, and the institution of emergency isolation procedures. Tuberculin skin tests/chest x-rays have been required for air travel from the Pacific islands to Hawaii for many years (PIHCP Referral Form). Since telemedicine, no patients with active TB are accepted. Now remote teleconsultation suffices for the diagnosis and treatment of Pacific Islanders with tuberculosis. The referral of an 18-year-old nursing student from Ebeye who was dying of a wasting disease is a case in point. We diagnosed adrenal tuberculosis (Addison’s disease) based on her exam, photographs, x-rays, labs, and ultra sound images. She was treated and restored to health (17). The uniqueness of the PIHCP allows for a better understanding of the epidemiology of both infectious and non-infectious diseases. Leptospirosis, prevalent in many of the islands of the Pacific reaches almost epidemic proportions in Kosrae State. Prior to the development of telemedicine, we summarized our experience with the diagnosis and treatment of acute leptospirosis with renal failure in children from the islands who were treated in Hawaii (18). Based on that experience and utilizing telemedicine, we are now able to remotely diagnose leptospirosis, recommend penicillin, fluid restriction, and supportive care. Children with leptospirosis no longer require medical evacuation. Similarly, children with tropical pyomyositis were sent urgently to TAMC, without approval, some unconscious and *in extremis*. Tropical pyomyositis, an acute pyogenic infection of muscle caused by *Staphylococcus aureus*, untreated, spreads, sepsis ensues, and many patients die. Since the introduction of telemedicine, island surgeons now know how to treat the condition aggressively with incision/drainage and anti-staphylococcal antibiotics. Teleconsultation allows these children to be treatment at home. Another great success of telemedicine is the surgical treatment of patients with perineal necrotizing fasciitis (Fournier’s gangrene). Using interactive PIHCP telemedicine, a surgical colleague in Majuro carefully documented (with intraoperative photographs) the extent and involvement of his patient’s infection. TAMC urology, surgery, and plastics consultants weighed in and recommended wide excision, diverting colostomy, burial of the testicle deep in his groin, and ultimately skin grafting. The surgeon presented a second patient and after a similar interaction and successful outcome, now Fournier’s gangrene cases are managed locally. Several years ago, two little girls fractured their femurs, one hit by a car (Majuro), the other fell out of a second story window (Pohnpei). Surgeons at both locations did what they could to provide traction without special equipment or orthopedic support. Each submitted his case to the PIHCP website (attached x-rays, photos, etc.) I was able to facilitate their consultations with TAMC’s pediatric orthopedist and we successfully managed both cases remotely at a great savings both emotionally and monetarily (19).

Some of my concerns for the future of the PIHCP include burgeoning telecommunications technology, advances in medical equipment/device development, drug/medication development, changes in medical care delivery, and changes in physician education. Complex military cyber security requirements threaten access to the PIHCP by distant providers. Hospitals in remote areas cannot afford or do not have access to advanced imaging technologies. Hospital laboratories are inadequate. Basic drugs, medications, and other pharmaceuticals are in short supply, unavailable, or unaffordable. Radiologists, pathologists, pharmacists, etc., are lacking. The delivery of health care in the US has evolved from hospital-based to clinic-based/day surgery outpatient care. Transvascular, endoscopic, and minimally invasive techniques have markedly changed modern health care practice in the US. Pace makers, implantable devices, shunts, pumps, etc., require specialized care and are contraindicated for use in Pacific Islanders, as follow up care is not guaranteed. On a more optimistic note, the US military has long been involved in disaster relief, civil affairs, medical civic action, and nation building. The PIHCP offers invaluable training, experience, and a better understanding of the people and conditions in the Developing World to students, residents, and staff prior to inevitable deployments to remote areas around the globe where they will be engaged in medical care similar to that provided patients from the USAPI. Cases from the PIHCP often challenge our basic observational, interpretive, integrative, and diagnostic skills. Caring for grateful Pacific Islanders with complex, advanced, medical conditions, restoring them to health, and returning them home as productive members of society is a truly rewarding experience. PIHCP telemedicine has bridged the gap between the distant Pacific and a major medical center.

## Conflict of Interest Statement

The author declares that the research was conducted in the absence of any commercial or financial relationships that could be construed as a potential conflict of interest.

## Supplementary Material

The Supplementary Material for this article can be found online at http://www.frontiersin.org/Journal/10.3389/fpubh.2014.00175/abstract
