# Supplementary material for: The Pacific Island Health Care Project
Source: Front Public Health. 2014 Oct 13;2:175. doi: 10.3389/fpubh.2014.00175 (PMC4195336; doi:10.3389/fpubh.2014.00175)
Supplement: Supplementary file 1 [file Presentation1.PDF]

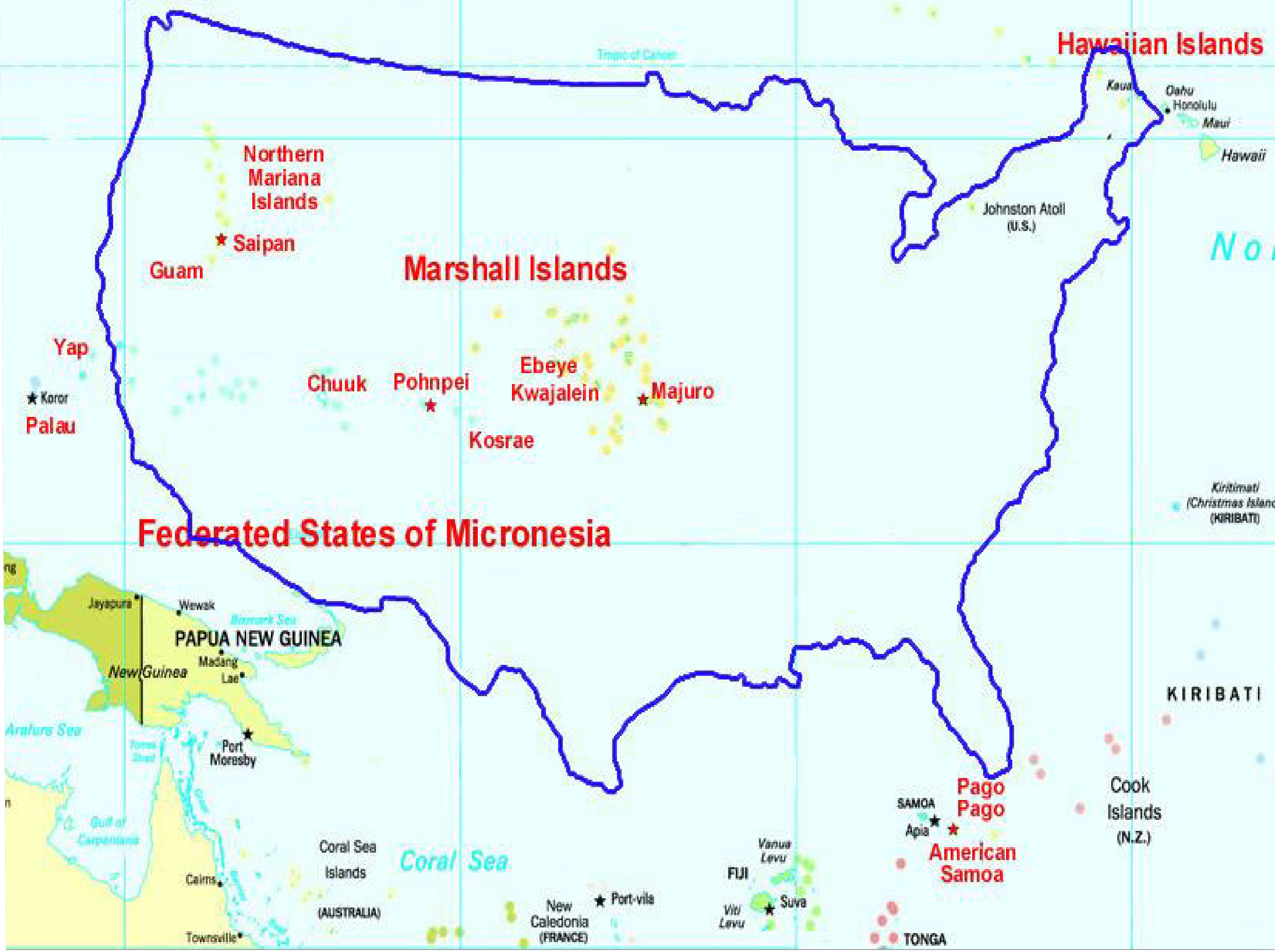

Supplementary Figure S1 - Map of the Pacific with US map superimposed

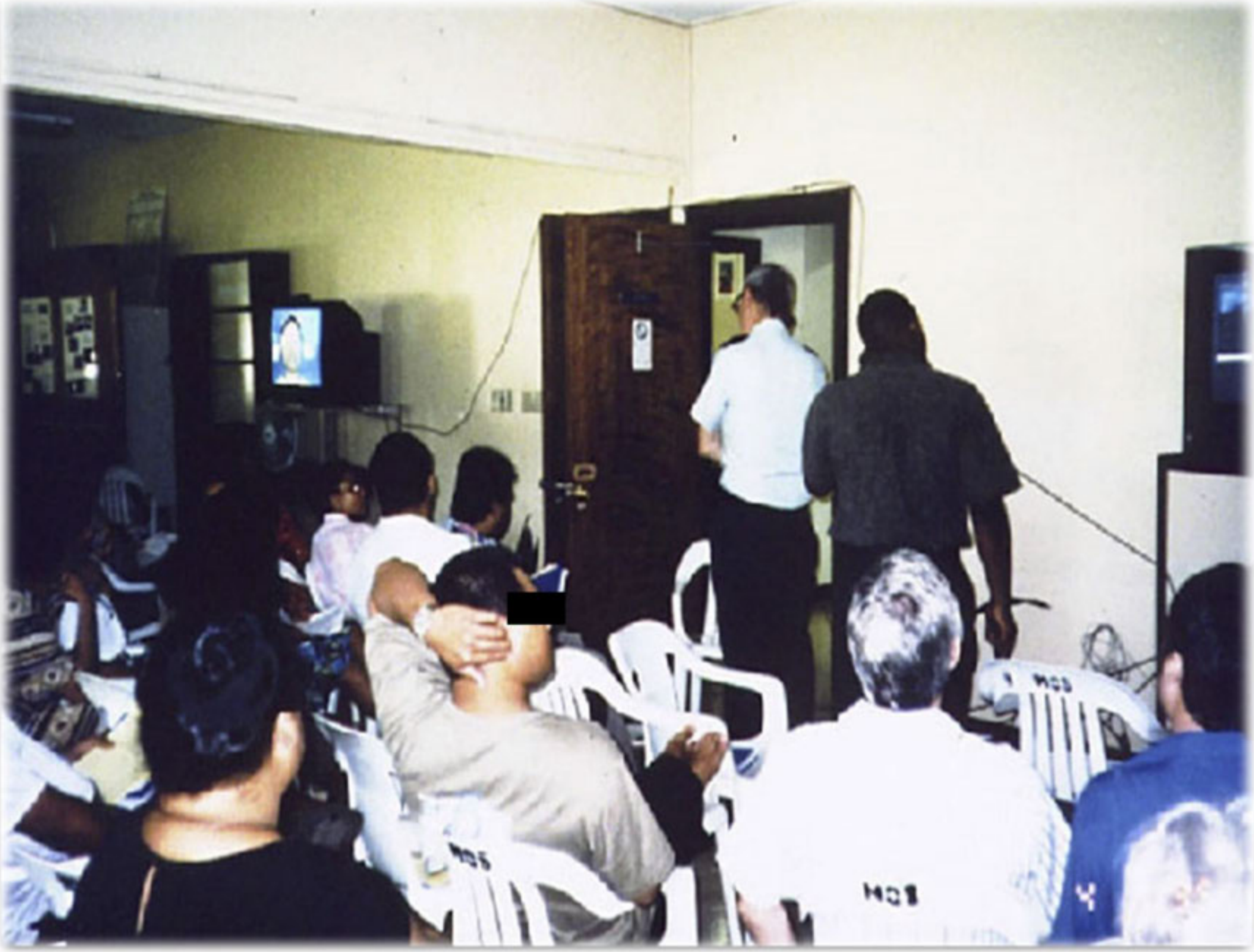

Supplementary Figure S2 - Author demonstrating Picasso phone at the inaugural meeting of the PBMA in Pohnpei State, FSM (1995)

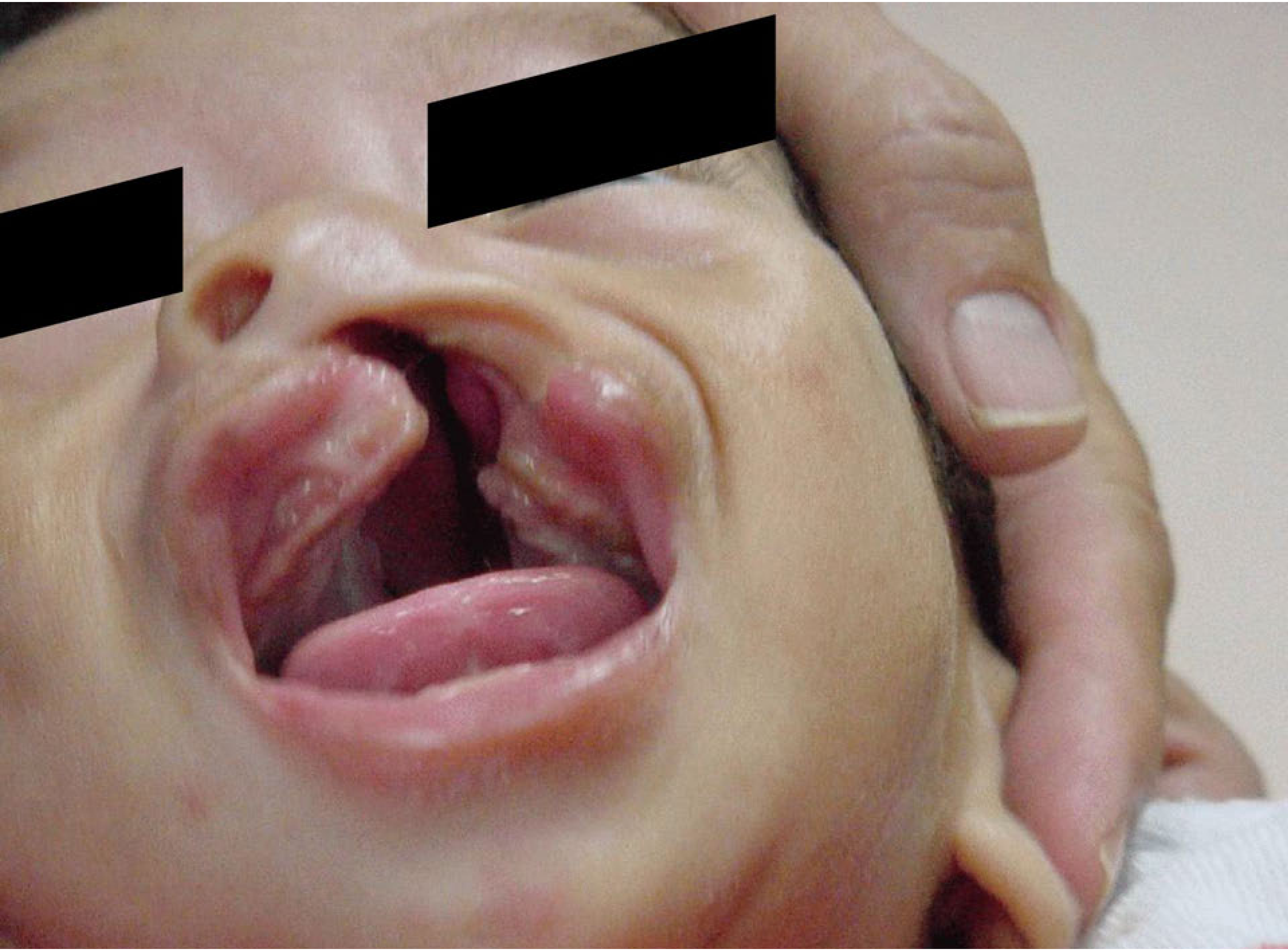

Supplementary Figure S3 - Infant from the Marshall Islands with cleft lip and cleft palate

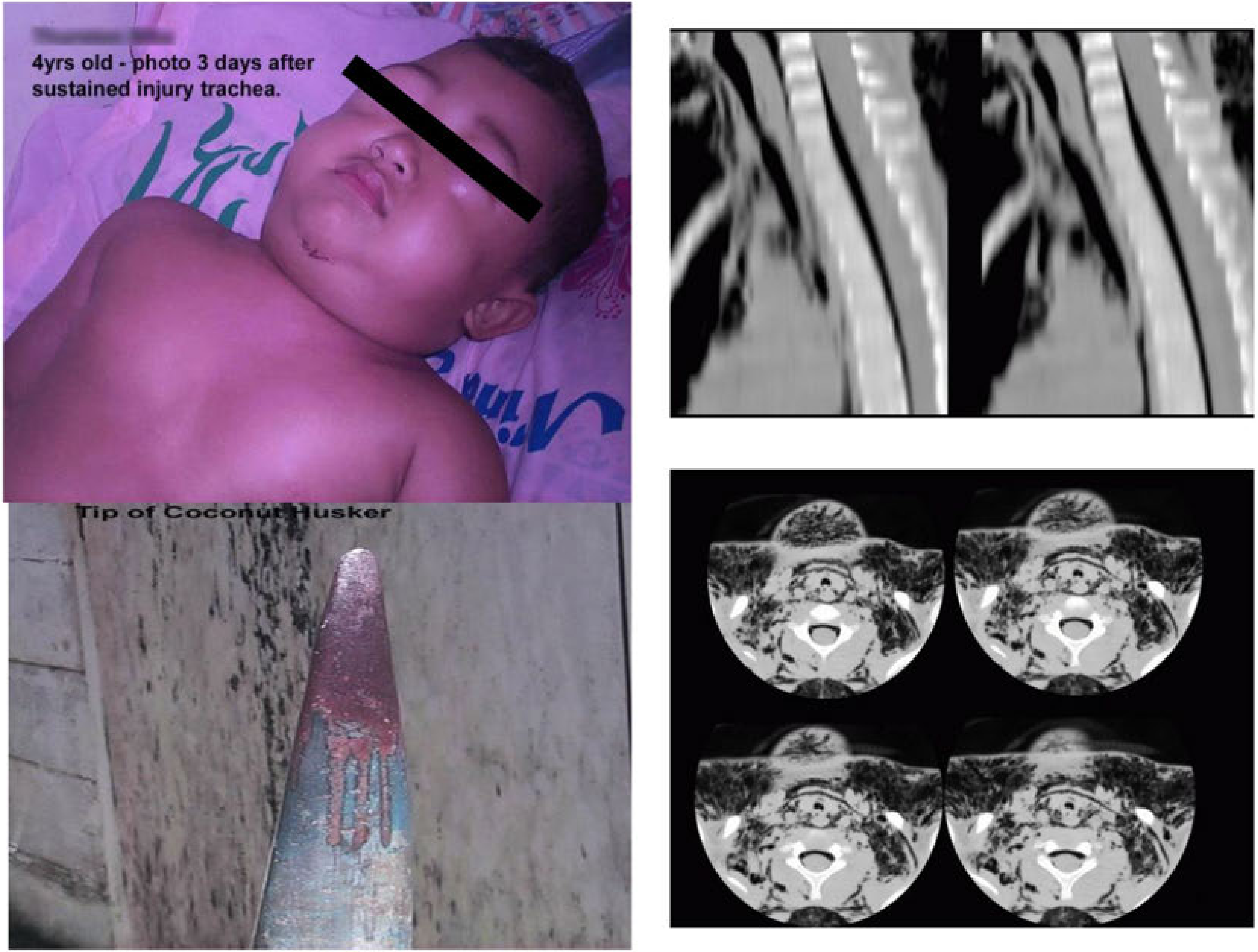

Supplementary Figure S4 - 4-year-old boy from Kosrae with traumatic laceration of his trachea Upper L, coconut husker, Lower L, child with massive subcutaneous emphysema, Upper R, lateral CT of spinal cord with intrathecal air, Lower R, cross sectional CT with diffuse emphysema

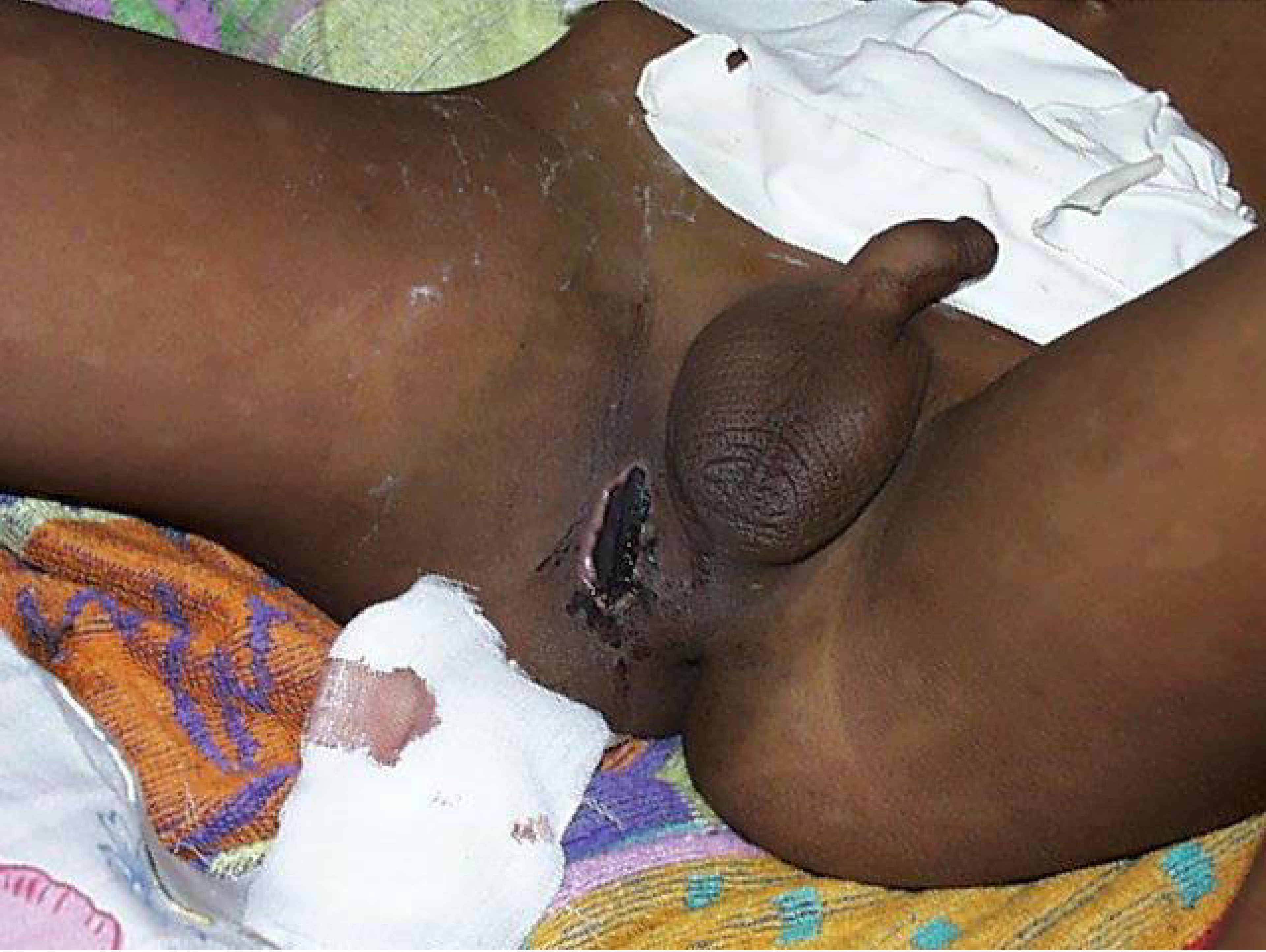

Supplementary Figure S5 - Deep laceration of a young Chuukese boy's perineum with laceration of his urethra, suprapubic cystostomy catheter in place

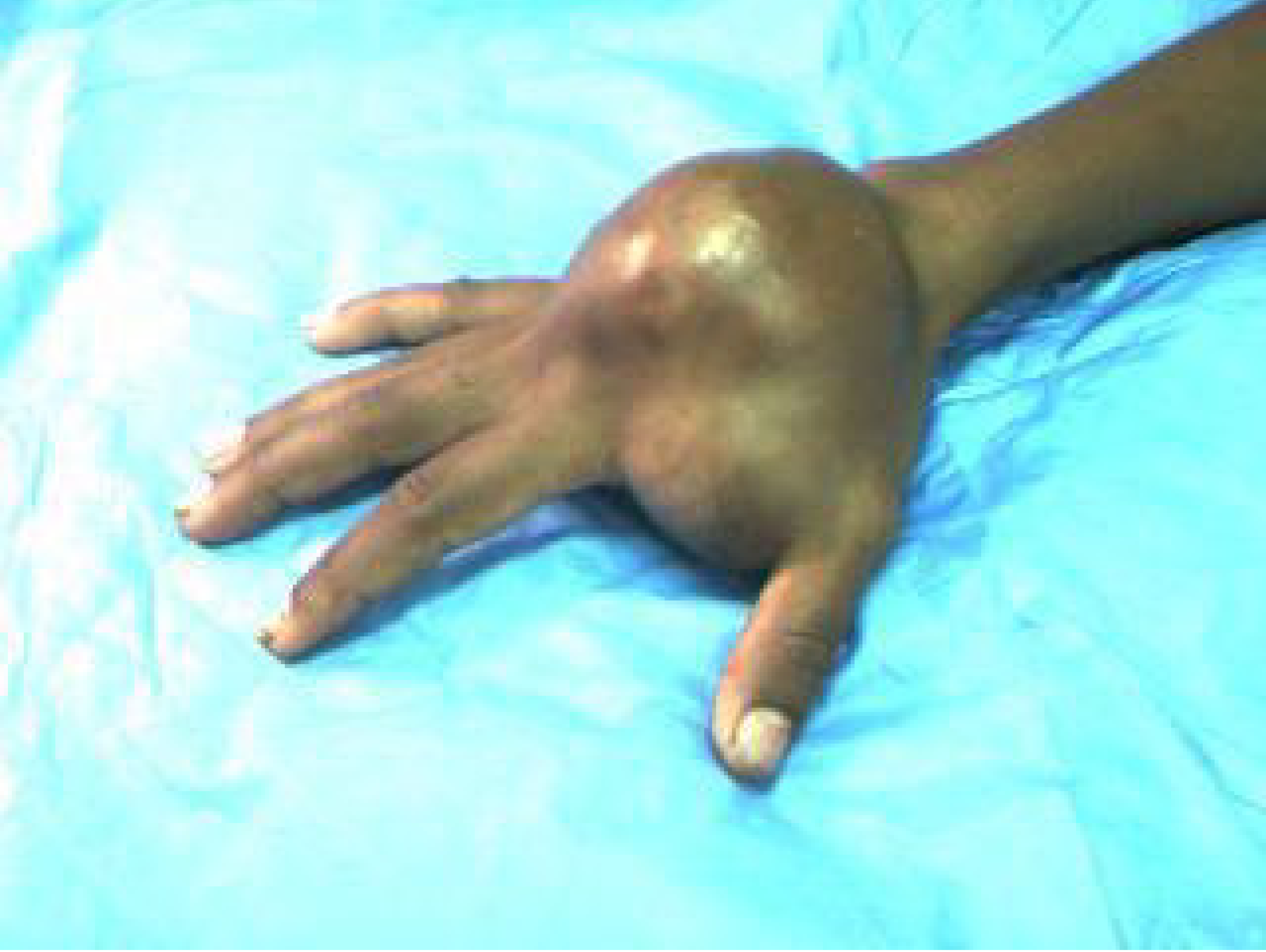

Supplementary Figure S6 - 22-year-old Yapese woman with rhabdomyosarcoma of her wrist/hand

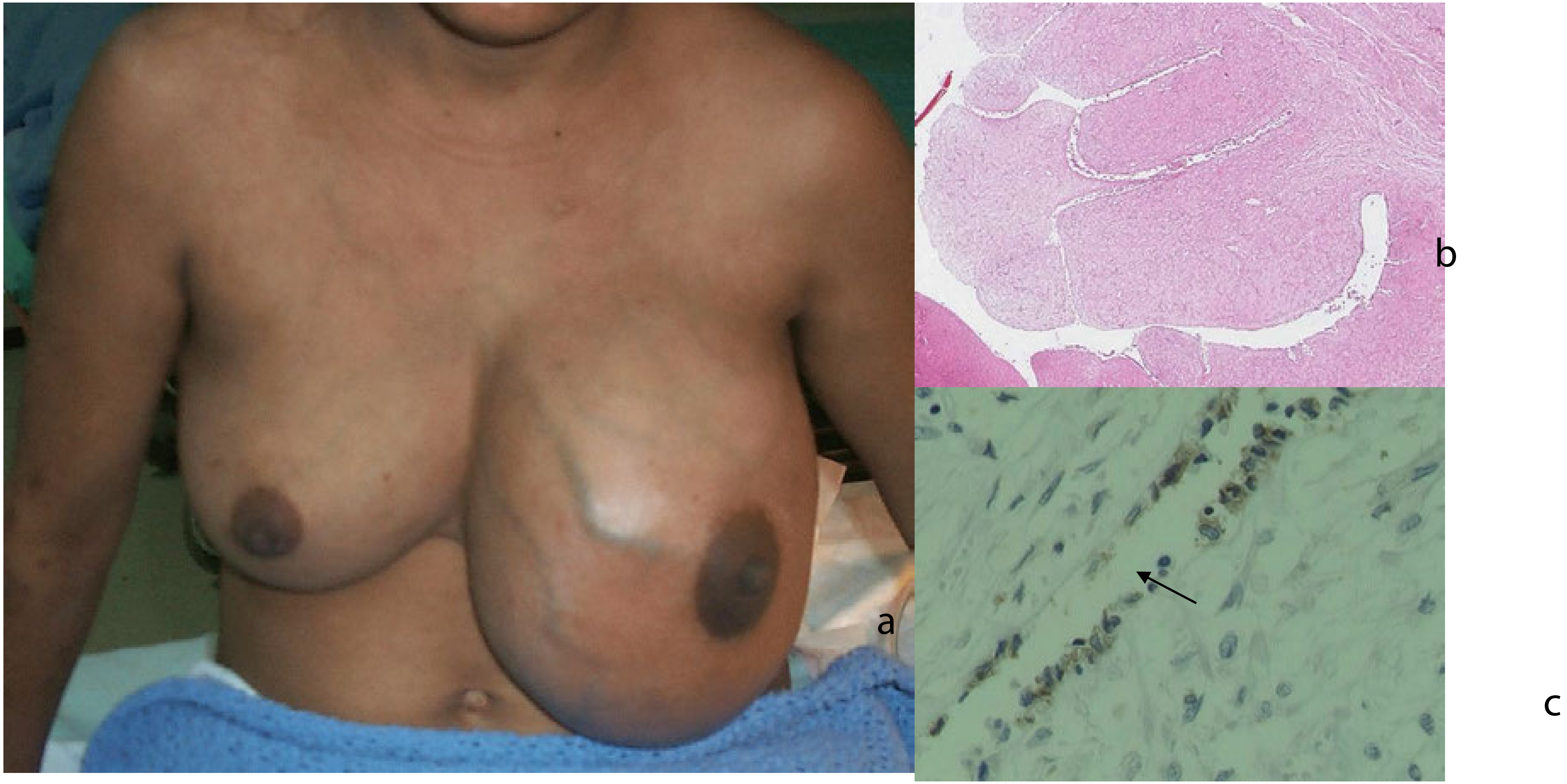

Supplementary Figure S7 - 34-year-old Marshallese woman with giant insulin secreting cystosarcoma phyllodes tumor (a) Upper R, H&E stained tumor section (b) and Lower R, ferritin tagged anti-insulin antibody stained (arrow) section (c)

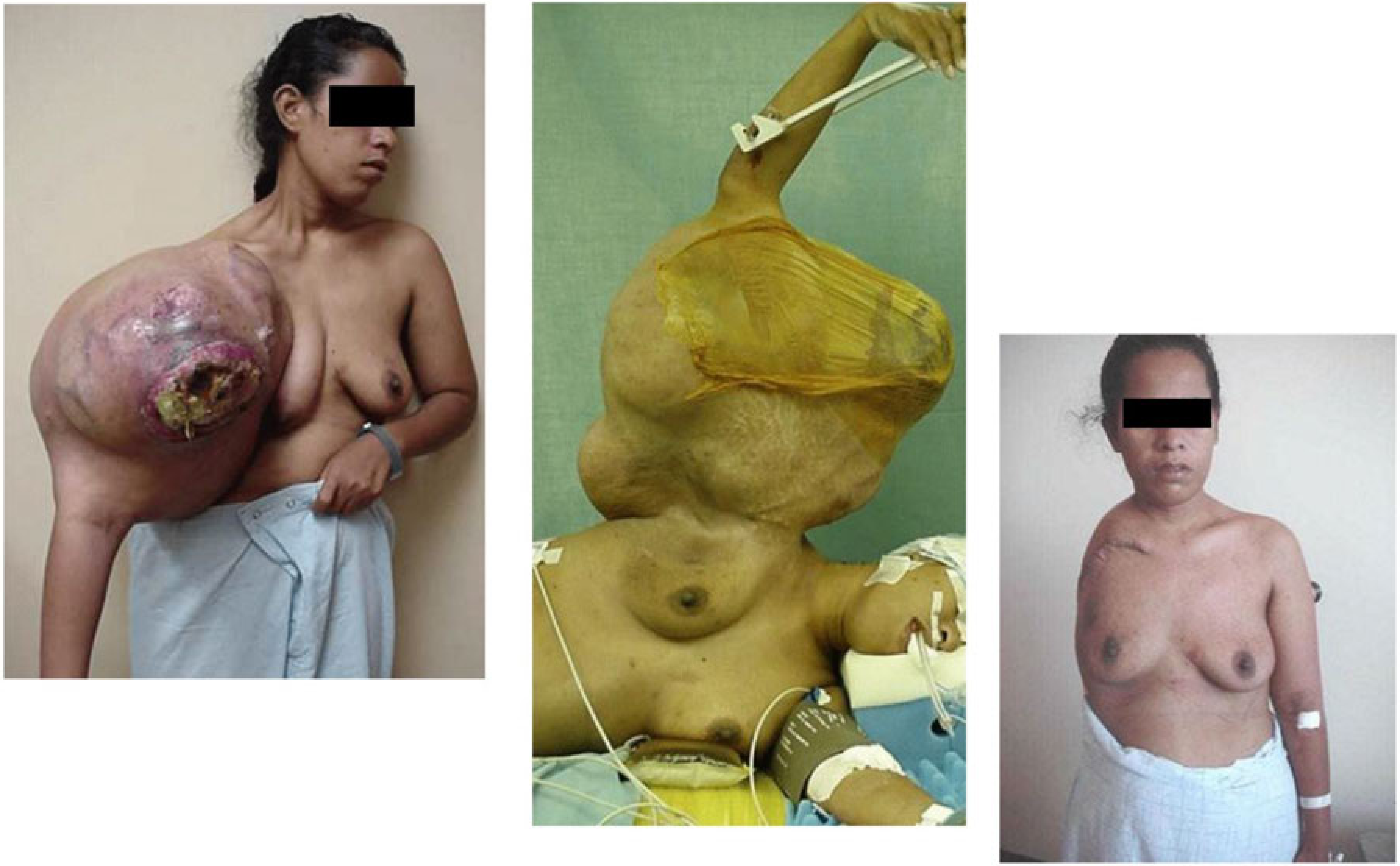

Supplementary Figure S8 - 28-year-old Pohnpeian woman with massive osteosarcoma of her R shoulder and upper arm (L), in the OR (middle), and after amputation (R)

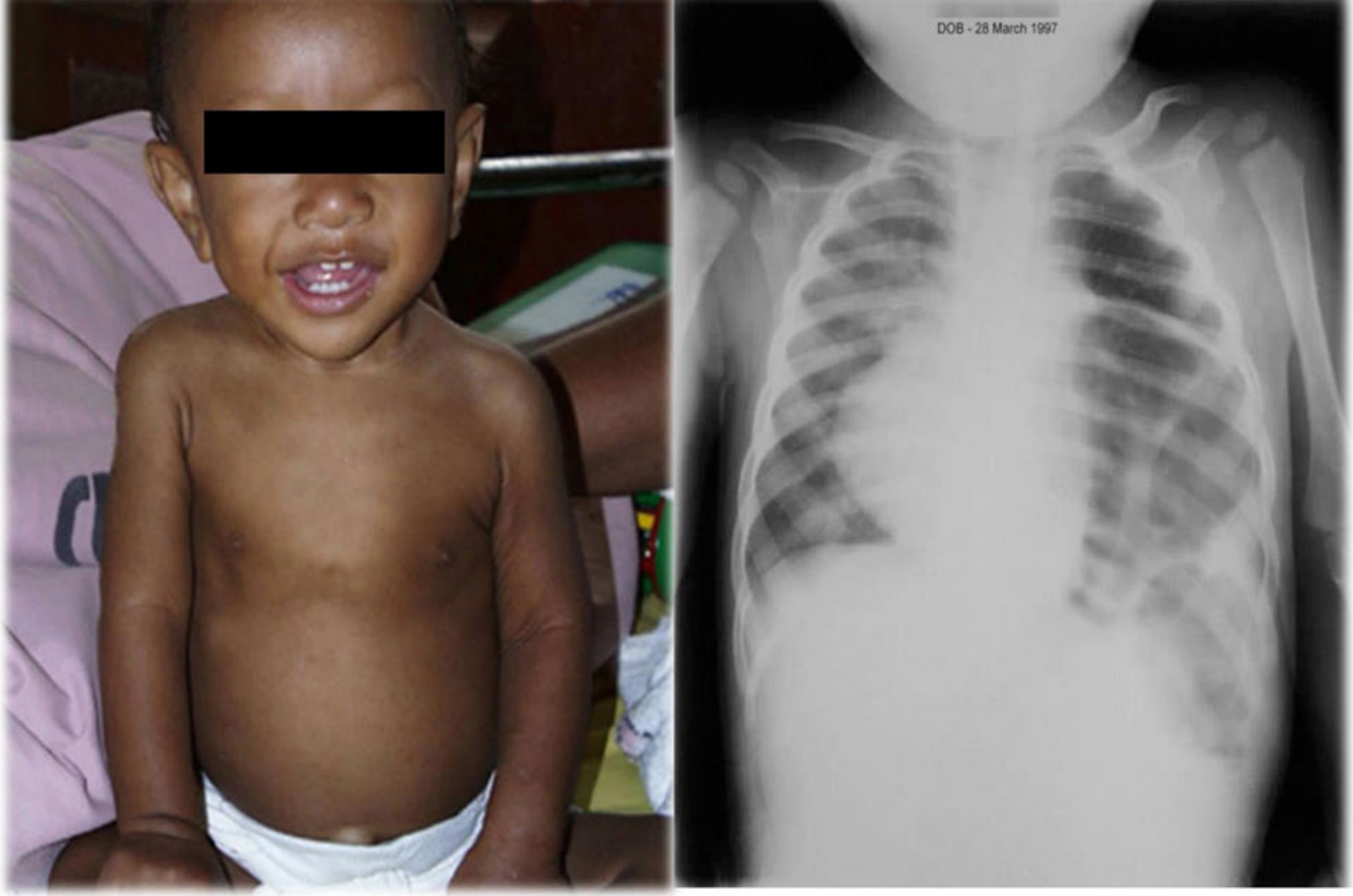

Supplementary Figure S9 - 18-month-old Pohnpeian boy with diaphragmatic hernia (bowel in left chest and heart shifted to right)

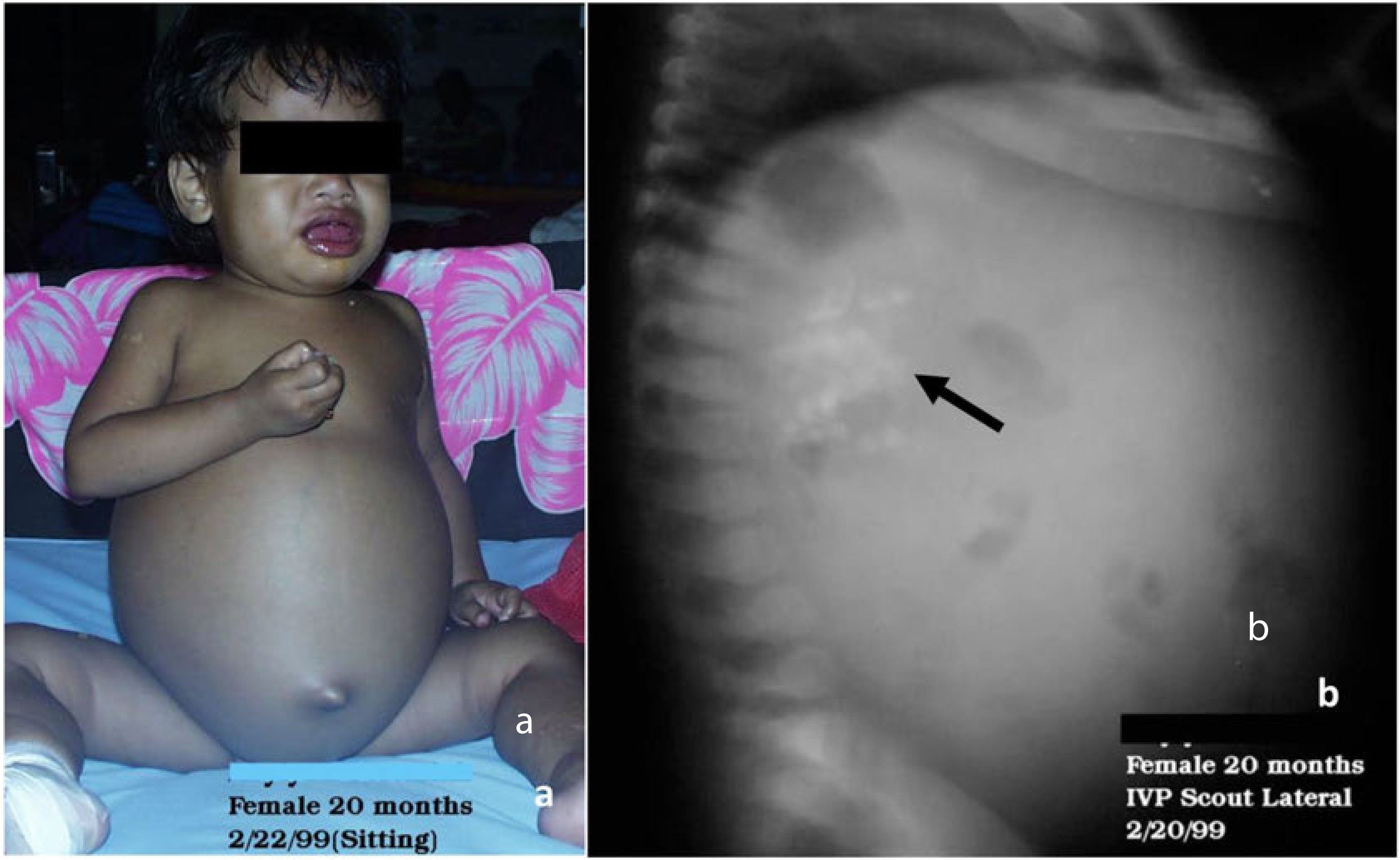

Supplementary Figure S10 - 20-month-old Pohnpeian girl with R/O Wilms tumor, teeth visualized on abdominal x-ray (arrow) confirm the diagnosis of teratoma

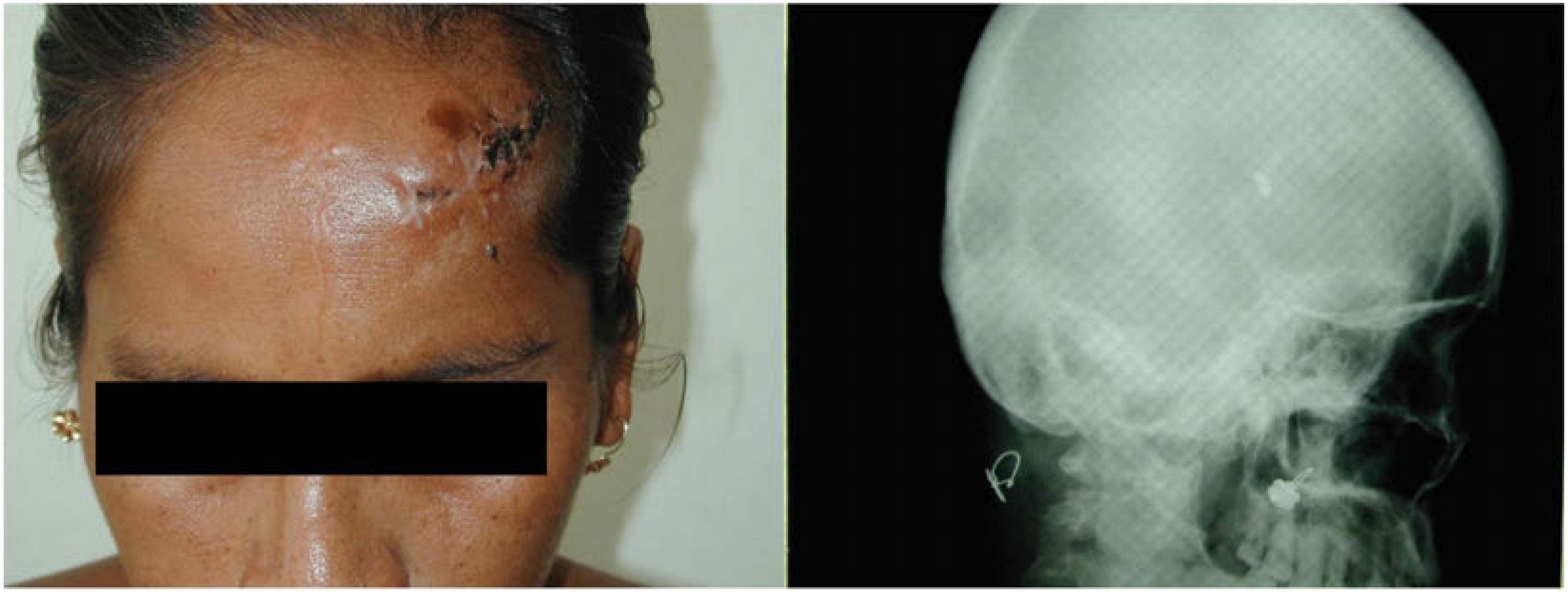

Supplementary Figure S11 - Pregnant Chuukese woman with gunshot wound to her head (L) and her skull x-ray with single .22 caliber bullet deep in her brain (R)

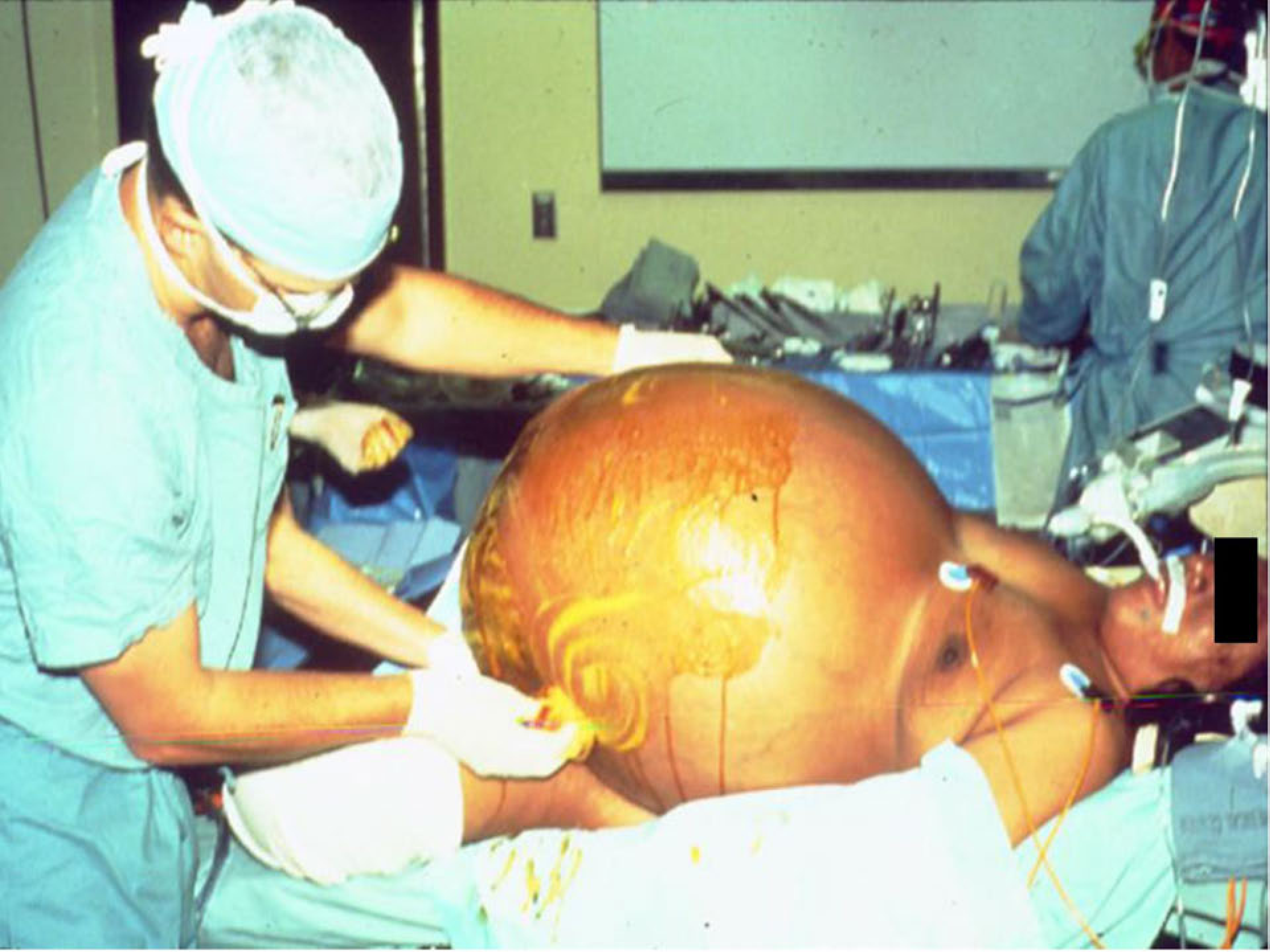

Supplementary Figure S12 - 38-year-old Marshallese woman with a massive serous cystadenoma of the ovary.
