# Supplementary material for: The Pacific Island Health Care Project
Source: Front Public Health. 2014 Oct 13;2:175. doi: 10.3389/fpubh.2014.00175 (PMC4195336; doi:10.3389/fpubh.2014.00175)
Supplement: Supplementary file 2 [file Data_Sheet1.DOCX]

**Table 1** Workstation Installations

Chuuk State Hospital, Weno, Chuuk State, FSM- February 1998

Belau State Hospital, Koror, Republic of Palau- March 1998

Pohnpei State Hospital, Kolonia, Pohnpei State, FSM-March 1998

Majuro Hospital, Majuro, Majuro Atoll, Republic of the Marshall Islands-March 1998

LBJ Tropical Medical Center, Pago Pago, American Samoa-January 1999

Kosrae State Hospital, Tofol, Kosrae State, FSM-September 1999

Ebeye Hospital, Kwajalein Atoll*, Republic of the Marshall Islands- September 1999

Yap State Hospital, Colonia, Yap State, FSM-January 2000

Commonwealth Health Center, Saipan, Northern Mariana Islands-January 2000

Guam Public Health Clinic, Mangilao, Guam-January 2001

*courtesy access provided the hospital at USAKA on Kwajalein Island

**Table 2** Distribution of Referrals to the PIHCP (4245 cases)

Jurisdiction* Number of cases (percent)

American Samoa 241 (5.7 %)

Chuuk State, FSM 427 (10.1 %)

CNMI 130 (3.1 %)

Ebeye/Kwajalein, RMI 567 (13.4 %)

Guam 34 (0.8 %)

Kosrae State, FSM 183 (4.3 %)

Majuro, RMI 1026 (24.2 %)

Palau 909 (21.4 %)

Pohnpei State, FSM 401(9.4 %)

Yap State FSM 327 (7.7 %)

*FSM, Federated States of Micronesia

*CNMI, Commonwealth of Northern Mariana Islands

*RMI, Republic of the Marshall Islands

**Table 3** PIHCP Demographics

Gender (4247 cases):

Females (girls, women) 2164 (50.9 %)

Males (boys, men) 2083 (49.1 %)

Age Distribution (4329 cases):

<18 years 1133 (26.2 %)

18-50 years 1840 (42.5 %)

>50 years 1356 (31.3 %)

**Table 4** Departmental referrals (based on 4246 cases)

Surgery 1714 (40.4 %)

Medicine 870 (20.5 %)

Pediatrics 544 (12.8 %)

Obstetrics/Gynecology 471 (11.1 %)

Other* 647 (15.2%)

* ENT, ophthalmology, urology, radiology, dental, psychiatry, pathology, etc.
